# Mealworm hydrolysate ameliorates dexamethasone-induced muscle atrophy via sirtuin 1-mediated signaling and Akt pathway

**DOI:** 10.1038/s41538-025-00432-9

**Published:** 2025-05-13

**Authors:** Sung-Min Kim, Jong-Yeon Kim, Eun-Min Jun, Varun Jaiswal, Eun-Jung Park, Hae-Jeung Lee

**Affiliations:** 1 Department of Food Science and Biotechnology, Gachon University, Gyeonggi-do, Republic of Korea; 2 Institute for Aging and Clinical Nutrition research, Gachon University, Gyeonggi-do, Republic of Korea; 3 Department of Food and Nutrition, Gachon University, Gyeonggi-do, Republic of Korea; 4 Department of Health Sciences and Technology, GAIHST, Gachon University, Incheon, Republic of Korea; 5 Gachon Biomedical Convergence Institute, Gachon University Gil Medical Center, Incheon, Republic of Korea

**Keywords:** Physiology, Health care

## Abstract

Loss of skeletal muscle mass and strength can result from various factors, including malnutrition, glucocorticoid usage, and diseases. The mealworm (*Tenebrio molitor* larvae) is an edible insect gaining popularity as an alternative protein-rich diet. Mealworms are expected to help alleviate muscle atrophy based on their rich, high-quality protein and peptide content, but it remains unclear whether mealworms ameliorate muscle loss. This study aimed to investigate the potential of mealworm hydrolysate (MH) in mitigating dexamethasone (DEX)-induced muscle atrophy and to elucidate the underlying mechanisms. MH ameliorates muscle atrophy by activating sirtuin 1 (SIRT1) and Akt, reducing muscle-specific RING finger protein-1 and atrogin-1 expression, and inhibiting apoptosis in DEX-treated C2C12 cells. Additionally, MH significantly increased the muscle mass, grip strength, and muscle fiber cross-sectional area by activating SIRT1 and Akt in DEX-treated rats. These findings suggest that MH has the potential in alleviating dexamethasone-induced muscle atrophy.

## Introduction

The predominant role of skeletal muscles is to provide mechanical mobility to the body and maintain posture based on a physiological process called excitation–contraction coupling^1^. Additionally, skeletal muscle functions as a metabolic and secretory organ, maintaining body temperature, absorbing and storing glucose, and interacting with other organs by secreting myokines^2,3^. The loss of muscle mass and function can be caused by various physiological and pathological mechanisms. In general, skeletal muscle is constantly broken down and synthesized, and skeletal muscle mass is maintained by balancing the protein degradation/synthesis rates. However, increased rates of muscle protein degradation or reduced rates of synthesis can result in the loss of skeletal muscle mass^4^. Lack of activity, aging, and disease are the potential causes of muscle atrophy.

Glucocorticoids (GCs), a type of steroid hormones, have long been used as useful agents to treat various medical conditions. However, stimulation of endogenous GC production contributes to muscle wasting under catabolic conditions, including diabetes and sepsis^5^. Similarly, high-dose or prolonged exposure to exogenous GCs, such as dexamethasone (DEX), results in the loss of muscle mass and function owing to their anti-anabolic and catabolic actions on skeletal muscle^6^.

Edible insects are a rich source of carbohydrates, proteins, fats, vitamins, and minerals, and have recently been recognized as healthy and environmentally friendly alternative protein and sustainable food sources^7^. At least 2,000 insect species, including beetles, mealworms, grasshoppers, crickets, termites, and ants have traditionally been consumed as food and feed^8^. Although insects were not considered food in Western countries until recently, dried mealworms were approved as the first safe insect food product and novel food at the EU level, according to European Food Safety Authority opinions, and were introduced to the market in 2021^9^. Recently, several edible insects have been evaluated not only as simple sources of nutrition but also as functional foods that provide physiologically beneficial effects^10^.

Enzymatic hydrolysis is an effective method for improving the functionality of proteins. Enzymatic hydrolysis using Alcalase, a widely used protease for protein hydrolysis, has been reported to improve the bioactive and techno-functional properties of insect proteins, including *Tenebrio molitor* larvae, *Gryllodes sigillatus*, and silkworm (*Bombyx mori*) pupae^11–13^. In addition, Yoon et al. reported that hydrolyzed mealworm protein exhibited stronger inhibitory effects against lipopolysaccharide-induced muscle loss in C2C12 cells, as well as greater antioxidant and anti-inflammatory activities compared to non-hydrolyzed mealworm protein^14^. Previous studies have reported that mealworms (*Tenebrio molitor* larvae) have antioxidant, anti-inflammatory, and immune-enhancing effects^15–17^. Although mealworm hydrolysate (MH), produced using Alcalase, is expected to help with muscle atrophy, the anti-atrophic effects of MH remain largely unexplored. In this study, we investigated the effects of MH on improving DEX-induced muscle atrophy and demonstrated the underlying mechanisms.

## Results

### Mealworm hydrolysate restores C2C12 cells from DEX-induced muscle atrophy and stimulates SIRT1 activity

**Fig. 1:**
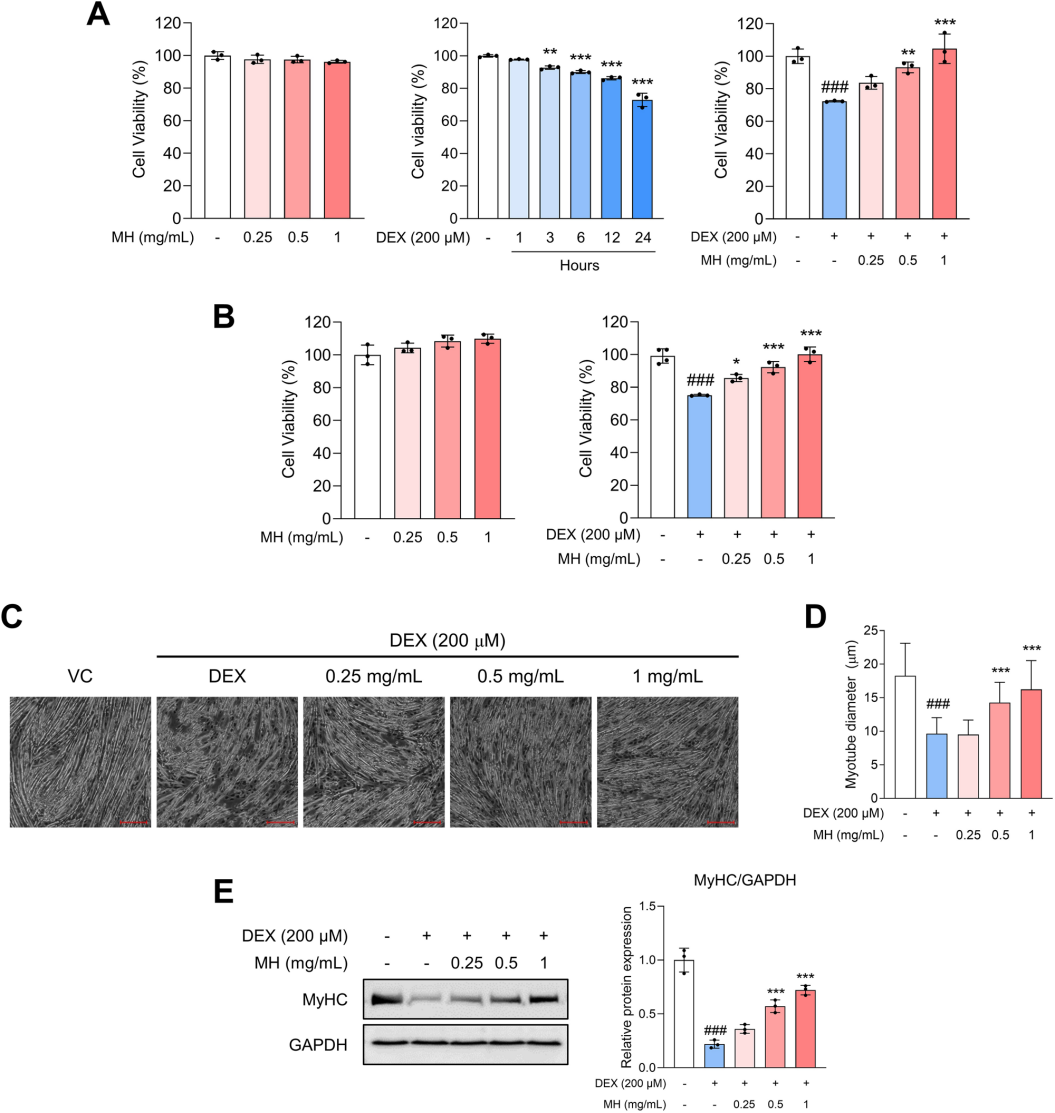
MH increases cell viability and ameliorates muscle atrophy in DEX-treated C2C12 cells. Cells were induced to differentiate starting 2 days after seeding until fully differentiated and then treated with DEX or MH alone for 24 h, or with DEX for 24 h followed by MH for an additional 24 h. **A** Cell viability in MH and/or DEX-treated C2C12 myotubes and **B** C2C12 myoblasts. **C**, **D** Morphology and diameter of myotubes. Magnification × 40. The scale bar indicates 200 µm. **E** Protein expression levels of MyHC. All experiments were repeated at least three times. Data are presented as the mean ± SD. ###*p* **<** 0.001, VC vs. DEX; *p **<** 0.05, **p **<** 0.01, ***p **<** 0.001, DEX vs. MH-treated groups. MH mealworm hydrolysate, DEX dexamethasone, VC vehicle control.

C2C12 myotubes were used to elucidate the effect of MH on DEX-induced muscle atrophy and its underlying mechanism. As shown in Fig. 1A, B, MH showed no cytotoxicity in C2C12 myotubes and myoblasts at concentrations of up to 1 mg/mL for 24 h. Additionally, the viability of myotubes treated with 200 μM DEX for 24 h was ~70% compared with the vehicle control and increased with MH treatment. Morphological observations showed that MH markedly increased myotube diameter (Fig. 1C, D). The protein expression levels of myosin heavy chain (MyHC), a major protein of myotubes and an indicator of late differentiation, were also increased, suggesting that MH improves protein degradation induced by DEX (Fig. 1E).

**Fig. 2:**
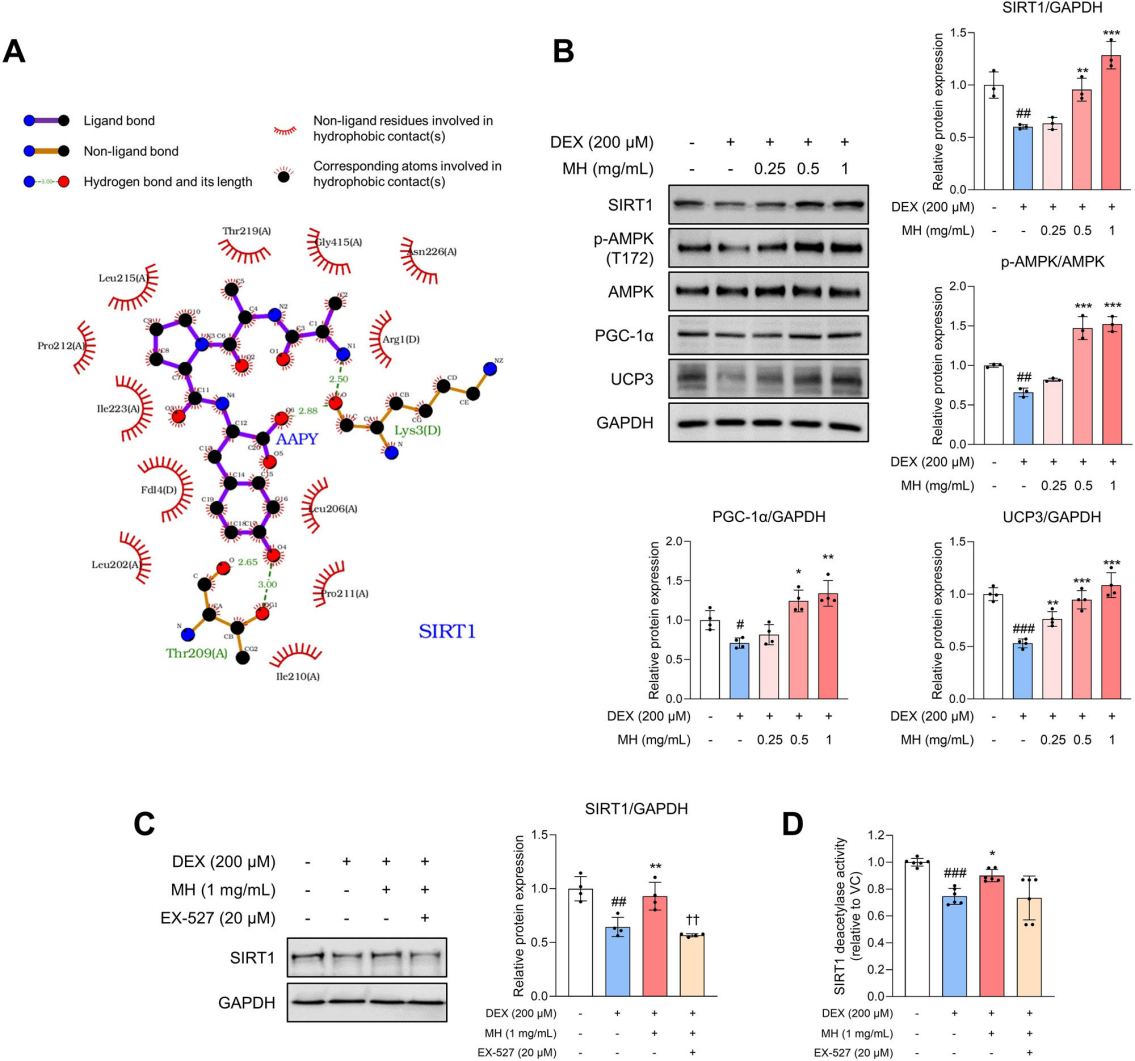
MH ameliorates muscle atrophy by stimulating SIRT1 activity in DEX-treated C2C12 cells. Cells were induced to differentiate starting 2 days after seeding until fully differentiated and then treated with DEX and MH for 24 h each. **A** Predicted protein-ligand interactions of AAPY (Ala-Ala-Pro-Tyr) with the SIRT1 through LigPlot^+^. **B** Protein expression and phosphorylation levels of SIRT1, AMPK, PGC-1α, and UCP3 depending on MH treatment concentration. **C**, **D** Protein expression levels and deacetylase activity of SIRT1 in the absence/presence of MH and EX-527 (SIRT1 inhibitor). All experiments were repeated at least three times. Data are presented as the mean ± SD. #*p* **<** 0.05, ##*p* **<** 0.01, ###*p* **<** 0.001, VC vs. DEX; *p **<** 0.05, **p **<** 0.01, ***p **<** 0.001, DEX vs. MH-treated groups; ††*p* **<** 0.01, MH-treated group vs. EX-527-treated group. MH mealworm hydrolysate, DEX dexamethasone, VC vehicle control, A alanine, P proline, Y tyrosine.

In addition, the molecular docking scores of MH-derived peptides with SIRT1, which is known to suppress muscle atrophy by inhibiting forkhead box O (FOXO) transcription factors and improving mitochondrial function, were evaluated. The seven types of peptides have a strong binding affinity with SIRT1, and AAPY (Ala-Ala-Pro-Tyr) had the strongest binding affinity at –14.01 kcal/mol (Table S1). Predicted interactions such as hydrogen bonding and hydrophobic interactions in the docked SIRT1–AAPY complex are shown in Fig. 2A. AAPY forms hydrogen bonds with residues Thr209 and Lys3. In the current study, Western blot analysis revealed that MH increased the protein expression of SIRT1, as well as the expression levels of AMP-activated protein kinase (AMPK), peroxisome proliferator-activated receptor γ coactivator 1α (PGC-1α), and uncoupling protein 3 (UCP3), which are closely associated with SIRT1 and play diverse roles in muscle, including mitochondrial biogenesis (Fig. 2B). Furthermore, MH increased the SIRT1 deacetylase activity in DEX-treated myotubes, which is important for regulating metabolic processes in the muscles. SIRT1 deacetylase activity increased by MH was attenuated in the presence of EX-527 (Fig. 2C, D).

### Mealworm hydrolysate regulates protein degradation and synthesis

**Fig. 3:**
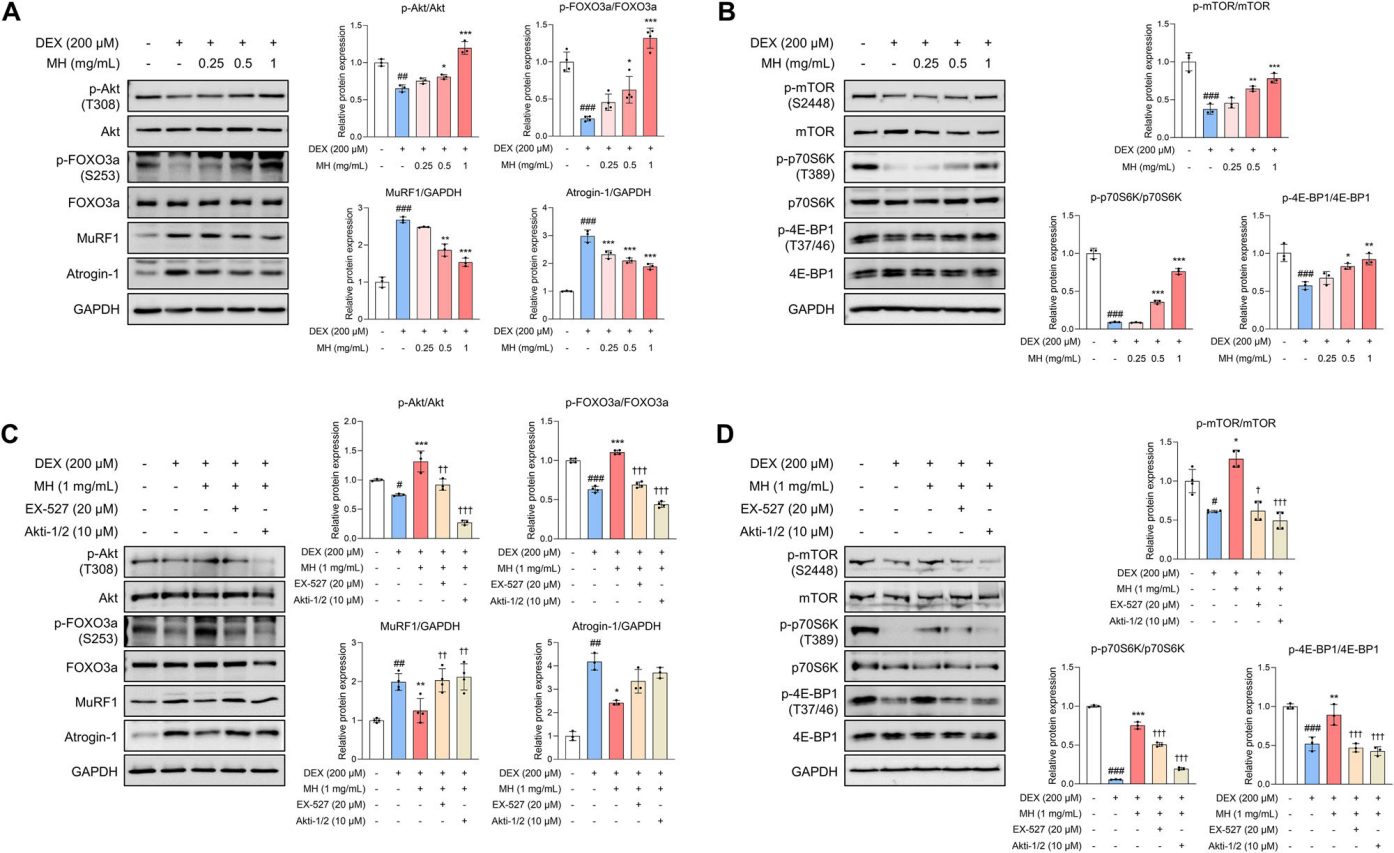
MH balances the expression levels of proteins regulating protein degradation and synthesis in DEX-treated C2C12 cells. Cells were induced to differentiate starting 2 days after seeding until fully differentiated and then treated with DEX and MH for 24 h each. **A** MH decreased the expression of MuRF1 and atrogin-1 by phosphorylating Akt and FOXO3a. **B** MH increased phosphorylation levels of mTOR, p70S6K, and 4E-BP1. **C**, **D** EX-527 (SIRT1 inhibitor) and Akti-1/2 (Akt inhibitor) disrupted the regulatory effects of MH on protein expression. All experiments were repeated at least three times. Data are presented as the mean ± SD. #*p* **<** 0.05, ##*p* **<** 0.01, ###*p* **<** 0.001, VC vs. DEX; *p **<** 0.05, **p **<** 0.01, ***p **<** 0.001, DEX vs. MH-treated groups; †*p* **<** 0.05, ††*p* **<** 0.01, †††*p* **<** 0.001, MH-treated group vs. inhibitor-treated group. MH mealworm hydrolysate, DEX dexamethasone, VC vehicle control.

The balance between protein degradation and synthesis that occurs constantly is important for maintaining skeletal muscle homeostasis. DEX treatment induced the expression of protein degradation markers while decreasing the expression of protein synthesis markers. MH inhibited the expression of muscle-specific RING finger protein-1 (MuRF1) and atrogin-1, which are molecular biomarkers of muscle atrophy, by increasing the phosphorylation of Akt and FOXO3a (Fig. 3A). Additionally, MH increased the phosphorylation levels of mammalian target of rapamycin (mTOR), p70 ribosomal S6 kinase (p70S6K), and eIF4E-binding protein 1 (4E-BP1), which promote protein synthesis (Fig. 3B). However, the regulatory effect of MH on the expression of aforementioned proteins was almost abrogated in the presence of EX-527 and Akti-1/2 (Fig. 3C, D). These results provide evidence that MH improves the imbalance between protein degradation and synthesis through Akt activation.

### Mealworm hydrolysate inhibits apoptosis and mitochondrial membrane potential changes

**Fig. 4:**
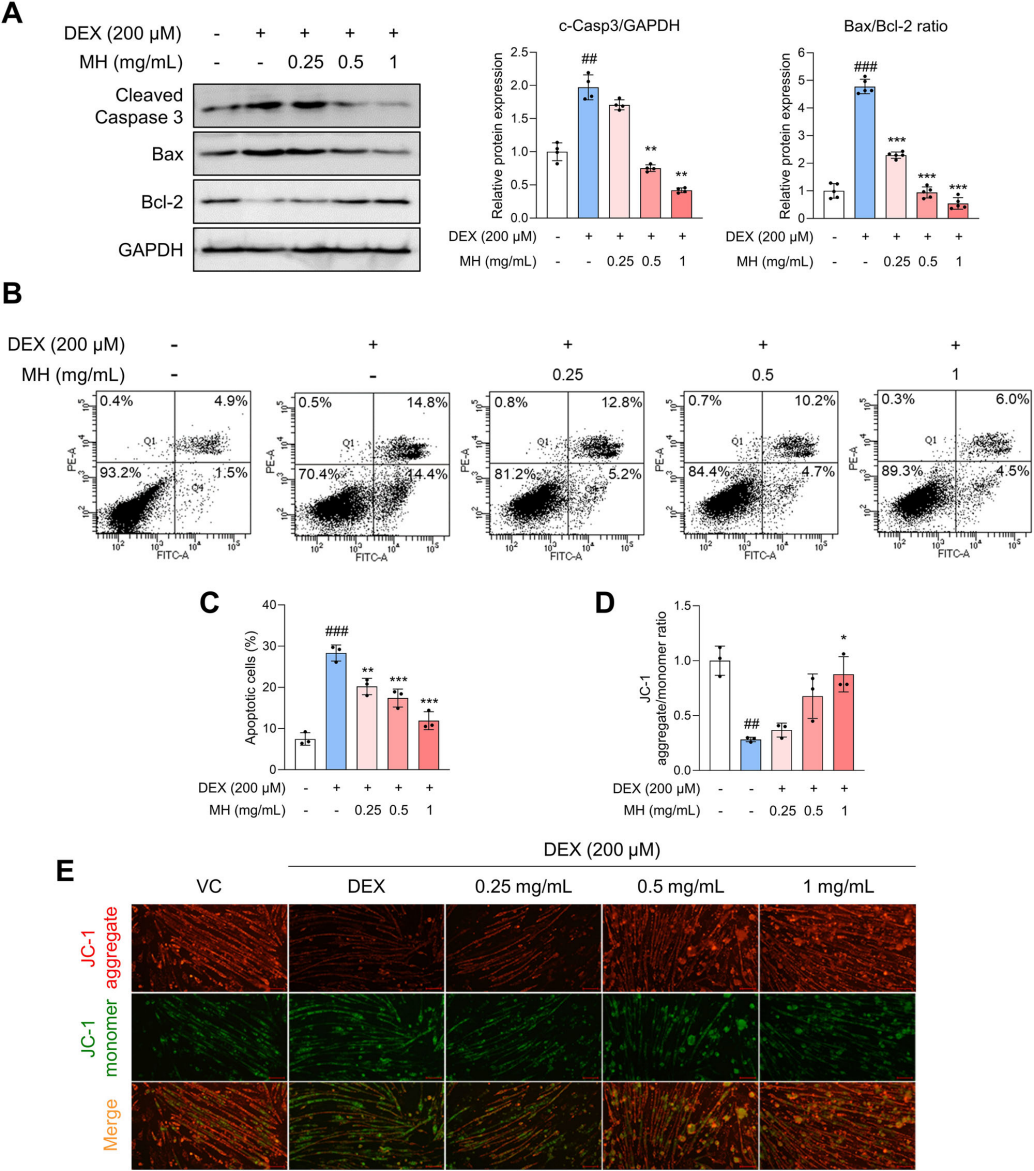
MH inhibits apoptosis and MMP changes in DEX-treated C2C12 cells. **A** Expression levels of apoptosis-related proteins in DEX-treated C2C12 myotubes. **B**, **C** Apoptotic cell distribution was analyzed using flow cytometry. Early apoptotic cells were marked as FITC positive/PI negative and late apoptotic cells were marked as FITC positive/PI positive. The percentage of apoptotic cells was calculated as the sum of the percentage of early and late apoptotic cells. **D**, **E** Effects of MH on JC-1 aggregate/monomer ratio in myotubes. JC-1 aggregates are displayed in red, whereas monomers are displayed in green. The ratio of red/green fluorescence intensity was quantified using ImageJ software. Magnification × 40. The scale bar indicates 200 µm. All experiments were repeated at least three times. Data are presented as the mean ± SD. ##*p* **<** 0.01, ###*p* **<** 0.001, VC vs. DEX; *p **<** 0.05, **p **<** 0.01, ***p **<** 0.001, DEX vs MH-treated groups. MH mealworm hydrolysate, DEX dexamethasone, VC vehicle control.

It has been reported that apoptosis increases during muscle atrophy caused by various conditions^18,19^. As shown in Fig. 4A, MH markedly reduced the expression of cleaved caspase-3 and the Bcl-2-associated X protein (Bax)/B-cell lymphoma 2 (Bcl-2) ratio. Additionally, we performed an Annexin V-FITC assay to determine the distribution of viable and apoptotic cells in the total cell population. The number of viable cells markedly decreased, and the number of apoptotic cells increased in cells treated with DEX compared with those treated with vehicle control. However, the number of early (Annexin V-FITC^+^/PI^–^) and late (Annexin V-FITC^+^/PI^+^) apoptotic cells was significantly reduced in the presence of MH (Fig. 4B, C). JC-1 can be used to detect loss of MMP within cells. A decrease in the red/green fluorescence intensity ratio was observed in cells treated with DEX, indicating depolarization of MMP, and MH alleviated the dissipation of MMP (Fig. 4D, E). Collectively, these results suggest that MH alleviates DEX-induced apoptosis and that the mitochondria-mediated apoptotic pathway is involved.

### Mealworm hydrolysate improves muscle mass and strength in DEX-treated rats

**Fig. 5:**
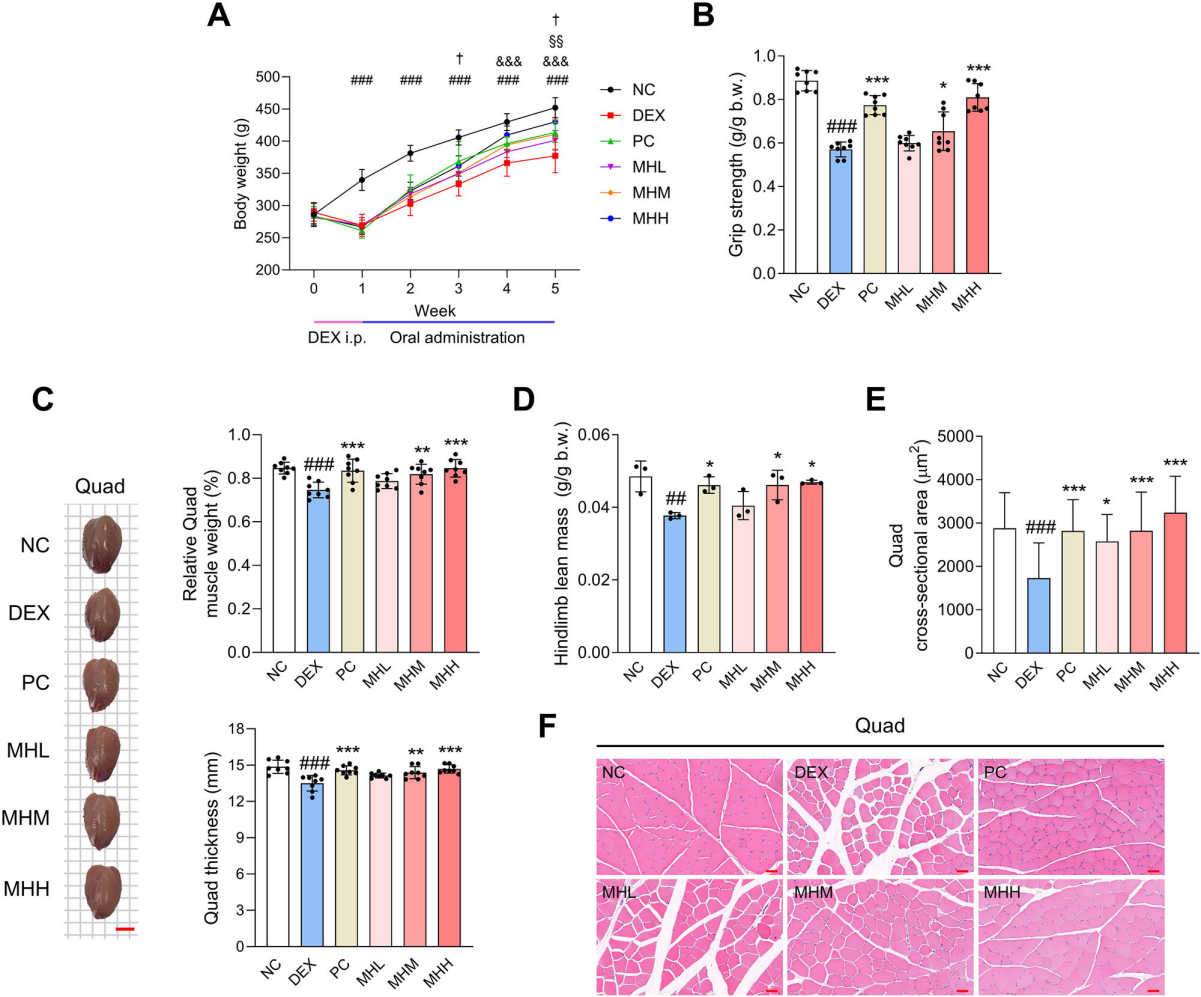
MH alleviates muscle atrophy in DEX-treated rats. Male SD rats were injected with DEX for 1 week, followed by oral treatment with oxymetholone (50 mg/kg/day) and MH (100, 200, and 400 mg/kg/day) for 4 weeks. **A** Body weight was measured once a week. **B** MH enhanced grip strength (normalized to body weight, *n* **=** 8). **C** Representative images, relative weight, and thickness of the Quad muscles (*n* **=** 8). The scale bar indicates 1 cm. **D** Hindlimb lean mass was measured using dual-energy X-ray absorptiometry (*n* **=** 3). **E**, **F** CSA of the Quad muscle fibers. Magnification × 200. The scale bar indicates 50 µm. Data are presented as the mean ± SD. ##*p* **<** 0.01, ###*p* **<** 0.001, NC vs. DEX; *p **<** 0.05, **p **<** 0.01, ***p **<** 0.001, DEX vs. PC and MH administration groups; †*p* **<** 0.05, DEX vs. PC; §§*p* < 0.01, DEX vs. MHM; & & &*p* < 0.001, DEX vs. MHH. MH mealworm hydrolysate, DEX dexamethasone, Quad quadriceps, CSA cross-sectional area.

We investigated whether MH supplementation could help overcome muscle atrophy in DEX-treated rats. The administration of DEX resulted in a significant decrease in body weight. The body weight of the MH administration groups gradually increased over the subsequent 4 weeks compared with that of the DEX group (Fig. 5A). Additionally, MH increased grip strength, which was decreased due to DEX treatment (Fig. 5B). Figure 5C, D show that the relative Quad weight, Quad thickness, and hindlimb lean mass increased in the MH administration groups. The relative weight and thickness of the Gas muscle also increased in the MH administration groups; however, no significant changes were observed in the Sol, TA, and EDL muscles (Fig. S1A–C). The CSA of the Quad muscle fibers was significantly smaller in the DEX group than that in the NC group, whereas it was reversed in the MH administration groups (Fig. 5E, F). Changes in the CSA of Gas muscle fibers showed a similar trend (Fig. S1D).

### Mealworm hydrolysate regulates muscle atrophy-related markers in DEX-treated rats

**Fig. 6:**
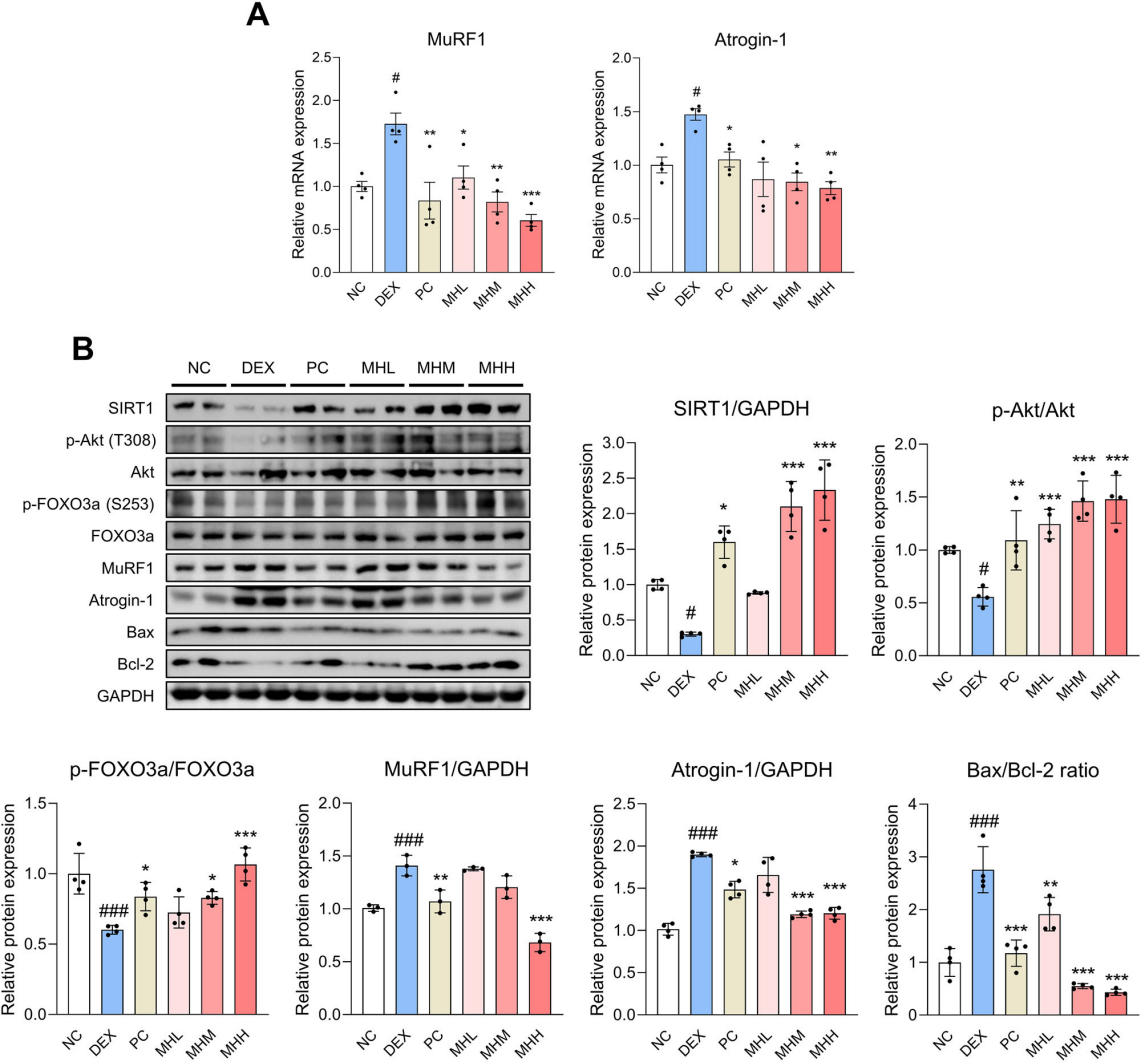
MH regulates molecular alterations in DEX-treated rats. Male SD rats were injected with DEX for 1 week, followed by oral treatment with oxymetholone (50 mg/kg/day) and MH (100, 200, and 400 mg/kg/day) for 4 weeks. **A** mRNA expression levels of MuRF1 and atrogin-1 in the Quad muscles. **B** Protein expression and phosphorylation levels of SIRT1, Akt, FOXO3a, MuRF1, atrogin-1, Bax, and Bcl-2 in the Quad muscles. Two lanes in each group represent two different rats. The expression levels of SIRT1, MuRF1, and atrogin-1 were normalized to GAPDH, whereas the expression of Bax and Bcl-2 was presented as the Bax/Bcl-2 ratio. All experiments were repeated at least three times. Data are presented as the mean ± SD. #*p* **<** 0.05, ###*p* **<** 0.001, NC vs. DEX; *p **<** 0.05, **p **<** 0.01, ***p **<** 0.001, DEX vs. PC and MH administration groups. MH mealworm hydrolysate, DEX dexamethasone, Quad quadriceps.

The mRNA expression levels of MuRF1 and atrogin-1 were increased in the Quad and Gas muscles of the DEX group, but downregulated in the MH administration groups (Fig. 6A and S1E). To further explore the mechanisms underlying the beneficial effects of MH on DEX-induced muscle atrophy in rats, we assessed the protein expression of markers related to muscle atrophy and apoptosis (Fig. 6B). DEX administration resulted in a decrease in the expression levels of SIRT1 and phosphorylation levels of Akt and FOXO3a; however, MH led to the overexpression of SIRT1, Akt, and FOXO3a, which in turn decreased the expression levels of MuRF1 and atrogin-1. Additionally, the reduced Bax/Bcl-2 ratio in the MH administration groups suggests that MH may have anti-apoptotic effects.

### Mealworm hydrolysate reduces inflammation and muscle damage in DEX-treated rats

Since glucocorticoids have been reported to enhance proinflammatory responses^20^, we assessed the serum levels of representative proinflammatory cytokines. Serum levels of IL-1β and TNFα tended to be lower in the MH administration groups. IL-1β secretion is increased by various stimuli in mice, including DEX treatment, and it causes muscle atrophy by inducing pyroptosis^21^. Therefore, the potential regulatory effects of MH on the secretion of proinflammatory cytokines may contribute to the improvement of DEX-induced muscle atrophy. Serum levels of lactate dehydrogenase (LDH) and creatine kinase (CK), which are markers of muscle damage, were also decreased in the MH administration groups (Table 1). No significant differences were observed in other measured parameters, including aspartate transaminase (AST) and alanine transaminase (ALT) (Table S2).

**Table 1:** Serum biochemical parameters in DEX-treated rats (*n* = 8)

	NC	DEX	PC	MHL	MHM	MHH
IL-1β (pg/mL)	17.82 ± 1.97	119.88 ± 148.42^#^	22.64 ± 6.07^*^	36.75 ± 26.17	24.61 ± 10.38^*^	25.97 ± 7.65^*^
TNFα (pg/mL)	3.86 ± 0.92	5.33 ± 1.96	3.66 ± 1.16	4.45 ± 0.99	3.10 ± 1.07^*^	2.91 ± 0.40^**^
LDH (U/L)	355.00 ± 131.01	743.67 ± 167.84^##^	400.33 ± 23.03^*^	580.50 ± 139.09	429.33 ± 74.77^*^	384.20 ± 81.12^*^
CK (U/L)	258.33 ± 22.30	433.75 ± 91.33^##^	275.40 ± 47.10^**^	309.20 ± 47.76^*^	291.33 ± 30.02^*^	261.75 ± 49.46^**^

*IL-1β* interleukin-1 beta, *TNFα* tumor necrosis factor alpha, *LDH* lactate dehydrogenase, *CK* creatine kinase.

Data are presented as the mean ± SD. #*p* < 0.05, ##*p* < 0.01, NC vs. DEX; *p < 0.05, **p < 0.01, DEX vs. PC and MH administration groups. *NC* normal control; *DEX* DEX-treated control; *PC* positive control, DEX treatment with 50 mg/kg oxymetholone; MHL, DEX treatment with 100 mg/kg MH; MHM, DEX treatment with 200 mg/kg MH; MHH, DEX treatment with 400 mg/kg MH.

## Discussion

Loss of muscle mass and function can be caused by various factors, including the adverse effects of drug treatment, malnutrition, and chronic disease. Muscle wasting affects physical abilities, reduces the quality of life, and contributes to increased mortality^22^. As the causes of muscle atrophy are diverse, many studies have been conducted to understand the molecular mechanisms and develop treatment strategies such as nutritional support, physical therapy, and drug therapy^23^. Although drugs such as anabolic medications and enzyme inhibitors have been shown to be effective in treating muscle atrophy, their clinical use remains limited as they can cause crucial adverse effects^24^. Therefore, the discovery of novel candidates that can ameliorate muscle atrophy is required.

Edible insects have been consumed for thousands of years as food and feed for humans and animals due to their high nutritional value. The advancement of science, including insect farming techniques and processing technologies, has led to edible insects becoming a promising alternative to fill nutrition gaps in communities and address food security threats globally^25^. Although meat and edible insects are excellent sources of nutrients, including proteins, unsaturated fatty acids, and minerals, edible insects have several advantages from an environmental perspective when raised compared with other animals. Insects require fewer resources such as feed, water, and land, and emit fewer greenhouse gases than standard farm animals^26^. Moreover, insects have an advantageous feed conversion efficiency and can be produced more efficiently as they are cold-blooded^27^. Despite the various benefits of edible insects, there are still some potential issues that should be considered. For example, consuming insects may be difficult to accept in cultures unfamiliar with the practice, and insect proteins may cause allergic reactions^28^. Enzymatic hydrolysis is an effective strategy to lower the psychological barrier to consuming insects, reduce or eliminate the allergenicity of insect proteins, and generate bioactive peptides^29^. Furthermore, it has been reported that protein hydrolysates are faster to digest and absorb in the body than intact proteins and that dietary amino acids tend to bind more rapidly to skeletal muscle proteins^30^. In this study, we propose that MH is a promising candidate for improving DEX-induced muscle atrophy.

Molecules and signaling pathways, including SIRT1, Akt/FOXO3a pathway, and mTOR/p70S6K pathway, are involved in skeletal muscle atrophy. The deacetylase SIRT1 suppresses muscle atrophy by reducing the expression of FOXO transcription factor and activating PGC-1α. Moreover, SIRT1 overexpression inhibits muscle atrophy and increases muscle mass by regulating the balance between protein degradation and synthesis^31^. Thus, stimulation of SIRT1 activation may be an effective approach for normalizing collapsed muscle mass and function. We revealed that MH induces SIRT1 activation, leading to the reduced expression of MuRF1 and atrogin-1. However, EX-527 abolished the effect of MH, confirming that its effect occurs through SIRT1-mediated signaling. In addition, we revealed that seven types of peptides derived from MH have a strong binding affinity for SIRT1 (Table S1). Among them, AAPY, which showed the strongest binding affinity, was found to interact with the Thr209 and Lys3 residues of SIRT1 through hydrogen bonds. Pinostilbene, a resveratrol analog, has been reported to interact with Thr209 and Lys3 of SIRT1 and significantly increase SIRT1 activity, supporting our findings that MH can increase SIRT1 activity^32^. Additionally, stigmasterol, which inhibits apoptosis and FOXO3a expression, has been reported to form a bond with Thr209, while rutin, which has antioxidant effects, binds to Lys3 of SIRT1. The interactions between these compounds and SIRT1 were similar to those formed by resveratrol, a representative SIRT1 activator^33,34^. Although we did not examine the effects of individual MH-derived peptides, the activating effect of MH on SIRT1 may be partially due to the various MH-derived peptides.

Increased expression of the muscle-specific E3 ubiquitin ligases MuRF1 and atrogin-1 results in muscle wasting by increasing protein degradation; therefore, high expression levels of MuRF1 and atrogin-1 are good markers for identifying muscle atrophy^35^. The transcription of MuRF1 and atrogin-1 is controlled by FOXO transcription factors, including FOXO3a. Akt phosphorylates FOXO3a at S253 and T32, allowing it to reside in the cytoplasm and thereby suppressing the transcriptional function of FOXO3a^36^. Therefore, Akt activation leads to the inhibition of MuRF1 and atrogin-1 expression, which alleviates muscle wasting. The Akt/mTOR pathway, which is involved in protein synthesis, is downregulated under atrophic conditions. Akt activates mTOR, which then stimulates protein synthesis by phosphorylating p70S6K and 4E-BP1^37,38^. Overall, Akt is important for performing the aforementioned roles, and MH increased the phosphorylation levels of Akt and FOXO3a while reducing the expression of MuRF1 and atrogin-1. The phosphorylation levels of mTOR, p70S6K, and 4E-BP1 also increased in the presence of MH. However, Akti-1/2 blocked the increase in the expression of Akt, FOXO3a, and mTOR, as well as the decrease in the expression of MuRF1 and atrogin-1 caused by MH, suggesting that MH regulates the balance between protein degradation and synthesis by participating in the Akt/FOXO3a and Akt/mTOR pathways.

It has been extensively reviewed that apoptosis is closely linked to the development of muscle atrophy due to various conditions, including glucocorticoids treatment^39,40^. A decrease in MMP caused by extracellular and intracellular stresses causes structural changes in the mitochondria, which in turn trigger the release of cytochrome c and activate caspase-3, leading to apoptosis^41,42^. MH increased the red/green ratio in JC-1 staining, indicating attenuation of MMP changes, and decreased the Bax/Bcl-2 ratio and cleaved caspase-3 expression, the active form of caspase-3. MH also markedly reduced the proportion of Annexin V-FITC-labeled cells. Further, we found that the Bax/Bcl-2 ratio in the Quad muscles was decreased. These results suggest that MH inhibits DEX-induced apoptosis, and the inhibition of apoptosis may contribute to the alleviation of muscle atrophy.

Kim et al. reported that *Gryllus bimaculatus* alleviates DEX-induced muscle atrophy by suppressing the expression of MuRF1, atrogin-1, and Smad2/3 while increasing the expression of Akt, mTOR, S6K, and MyHC^43^. Additionally, Lee et al. reported that *Protaetia brevitarsis* protein extract, which has antioxidant activity, exhibits a protective effect on C2C12 cells by inhibiting apoptosis and MMP reduction induced by oxidative stress^44^. These findings suggest that not only mealworms but also various species of edible insects may have beneficial effects on muscle atrophy. Meanwhile, obesity is another factor that can contribute to muscle atrophy^45^. Mealworms have been reported to reduce body fat in aged obese mice, whereas *Gryllus bimaculatus*-containing diets showed no effect on body fat in a high-fat diet-induced obesity mouse model^43,46^. Although differences in study design should be considered, these findings indicate that the physiological benefits provided by different insect species may vary.

According to well-organized review papers, insects are generally rich in high-quality protein, and several insect species have been reported to possess various beneficial effects, including antioxidant and anti-inflammatory properties^10,47^. Therefore, a considerable number of insect species are expected to have beneficial effects on muscle atrophy, but limited studies have been conducted compared to the vast number of known insect species. Considering the diversity of causes of muscle atrophy, the unique features of each insect species, various processing methods, and the types of species accepted in different countries, it would be a worthwhile study to investigate the effects of other edible insect species on muscle atrophy.

In conclusion, MH alleviated DEX-induced muscle atrophy by activating SIRT1 and Akt. SIRT1 and Akt inhibitors abolished the decrease in the expression of MuRF1 and atrogin-1 resulting from MH, demonstrating that the beneficial effects of MH were mediated through SIRT1-mediated signaling and Akt pathway. The inhibitory effect of MH on apoptosis could also contribute to improving muscle atrophy. These findings suggest that MH has the potential to be a functional agent for mitigating muscle atrophy. Further well-designed clinical trials are needed to ultimately apply MH to patients with muscle atrophy.

## Methods

### Mealworm hydrolysate preparation

MH was supplied by Hanmi Nutrition Inc. (Gyeonggi-do, Korea). MH was produced in accordance with standard production procedures. Briefly, powdered dried mealworm was hydrolyzed using Alcalase® (1% w/w; Novozymes, Bagsvaerd, Denmark) at 50 °C. The sludge was removed via centrifugation at 14,000 rpm with tubular centrifuge. The end product (MH) was obtained through sterilizing, cooling, concentrating, and freeze-drying. The manufacturing flows are summarized in Fig. S2. The peptide profiling by liquid chromatography-mass spectrometry peaks is shown in Fig. S3, and the sequence is presented in Table S3.

### Mealworm hydrolysate-derived peptide profiling by liquid chromatography-mass spectrometry analysis

An Ultimate 3000 system equipped with coupled with quadrupole time-of-flight mass spectrometry (HPLC-Q-TOF-MS, Bruker Daltonics, 255748 Germany) was used for the chromatographic separation and peptide profiling. Separation was carried out on a Zorbax eclipse plus C18 (100 mm × 3.0 mm, 1.8 μm; Agilent, USA) at 30 °C, with an injection volume of 5 μL. The mobile phases consisted of 0.1% formic acid (v/v) (A) and 0.1% acetonitrile (v/v) (B). The flow rate was 0.3 mL/min, and the gradient program was as follows: 2–2% B (0–2 min), 2–30% B (2–30 min), 30–98% B (30–31 min), and 98–98% B (31–36 min), 98–2% B (36–37 min), 2–2% B (37–45 min). The content of the AAPY (Ala-Ala-Pro-Tyr) peptide in the MH was quantified as 1.49 ± 0.14 mg/g.

### Cell culture, differentiation, and viability assay

Mouse C2C12 myoblasts were obtained from American Type Culture Collection (Manassas, VA, USA) and grown in Dulbecco’s modified Eagle medium (Corning, Manassas, VA, USA) supplemented with 10% fetal bovine serum (FBS; Gibco, Grand Island, NY, USA) and 1% antibiotic-antimycotic (Gibco, Grand Island, NY, USA) in a 5% CO_2_ incubator at 37 °C. When the cells reached 90–100% confluence, the growth medium was replaced with a differentiation medium containing 2% horse serum (Sigma-Aldrich, St. Louis, MO, USA) instead of 10% FBS. The differentiation medium was changed every 2 days until the cells were fully differentiated and formed myotubes. Cells were treated with 200 μM DEX (Sigma-Aldrich, St. Louis, MO, USA) for 24 h and then incubated with MH in the presence or absence of EX-527 (SIRT1 inhibitor; Selleckchem, Houston, TX, USA) and Akti-1/2 (Akt inhibitor; Sigma-Aldrich, St. Louis, MO, USA) for 24 h. MH, DEX, EX-527, and Akti-1/2 were dissolved in phosphate buffered saline (PBS) and used, while the vehicle control was treated with an equal volume of PBS. Cell viability was evaluated using the Cell Counting Kit-8 assay (Dojindo Laboratories, Kumamoto, Japan) according to the manufacturer’s instructions. The diameters of at least 20 myotubes were measured in three different fields using ImageJ software (National Institutes of Health, USA).

### Sirtuin 1 (SIRT1) deacetylase activity assay

SIRT1 deacetylase activity was assessed using a SIRT1 fluorometric assay kit (Abcam, Cambridge, UK), according to the manufacturer’s protocol. Briefly, a nuclear extraction buffer was prepared as suggested in the datasheet and used to isolate the nuclear fractions from C2C12 cells. bicinchoninic acid (BCA) assay (Takara Bio Inc., Kusatsu, Shiga, Japan) was used to quantify the protein concentration in the nuclear fraction. Fluorescence intensity was measured at 340 nm excitation and 460 nm emission using a GloMax® Discover microplate reader (Promega, Madison, WI, USA).

### Annexin V-FITC apoptosis detection assay

Cell apoptosis was detected using an Annexin V-FITC/propidium iodide (PI) apoptosis detection kit (Abcam, Cambridge, UK), according to the manufacturer’s instructions. Briefly, cells treated with DEX and MH were trypsinized, washed, and resuspended in 500 μL of binding buffer. The cells were subsequently stained with Annexin V-FITC and PI for 5 min in the dark at room temperature. Cell distribution was analyzed using a FACSCanto II flow cytometer (BD Biosciences, San Jose, CA, USA).

### Mitochondrial membrane potential (MMP) measurement

The MMP was evaluated using the JC-1 dye (Invitrogen, Carlsbad, CA, USA). Briefly, cells were stained with JC-1 dye at a final concentration of 2 μM for 15 min at 37 °C in the dark. Fluorescence images were obtained using a fluorescence microscope and fluorescence live imaging camera (Korea Lab Tech., Gyeonggi-do, Korea). Fluorescence intensity was calculated using ImageJ software (National Institutes of Health, USA). The red/green (JC-1 aggregate/monomer) intensity ratio reflects the changes in MMP.

### Molecular docking analysis

Molecular docking analysis was carried out as described previously^48^. Briefly, the X-ray crystal structure of SIRT1 was taken from protein data bank database (PDBID: 5BTR)^49^. The resveratrol and water molecules present in the crystal structure were removed from the target receptor protein. Protein and peptide ligand preparation which includes structure editing adding charges and hydrogen atoms was carried out through UCSF-Chimera^50^ and AutoDockTool kit^51^. The binding pocket for ligand docking was defined to cover the entire binding site of the target receptor protein and the surrounding residues using AutoDockTool kit. Finally, the docking was carried out using AutoDock4.2. Docking poses of peptides were ranked according to estimated free energy of binding of ligands. Interaction studies of protein-ligand in the docked complexes were conducted through LigPlot^+^ software with an academic license^52^. The interactions between ligand and proteins were also manually verified through manual visualization of docked complexes in UCSF Chimera.

### Animal procedures

All male Sprague–Dawley rats were obtained from Samtako Bio Korea (Osan, Korea) and housed under constant conditions of 20–25 °C temperature and 50–55% humidity on a 12 h light-dark cycle. Rats were given a 1-week acclimation period and fed rodent chow (Purina Korea, Gyeonggi-do, Korea) and water *ad libitum*. Six-week-old rats were randomly divided into six groups (*n* = eight rats per group) based on body weight as follows: (i) NC, normal control; (ii) DEX, DEX-treated control; (iii) PC, positive control, DEX treatment with 50 mg/kg oxymetholone; (iv) MHL, DEX treatment with 100 mg/kg MH; (v) MHM, DEX treatment with 200 mg/kg MH; and (vi) MHH, DEX treatment with 400 mg/kg MH. The sample size was determined based on previous studies with similar experimental designs and expected effect sizes^53,54^. To induce muscle atrophy, all rats, except the NC group, were intraperitoneally injected with DEX for 7 days. Body weight was measured daily in this period and the injection amount of DEX was adjusted to 500 μg/kg depending on body weight. The DEX, PC, and MH administration groups were orally administered sterile saline solution, oxymetholone, and MH for 4 weeks, respectively. The NC group was administered a sterile saline solution during the entire experimental period. Oxymetholone (17β-hydroxy-2-(hydroxymethylene)-17-methyl-5α-androstan-3-one; Celltrion Pharm Inc., Jincheon, Korea), a 17α-alkylated anabolic-androgenic steroid, was employed as a treatment agent for the PC group as it has been reported to increase muscle mass and strength^55^. Body weight was measured weekly. To measure hindlimb lean mass, scans were performed on anesthetized rats using dual-energy X-ray absorptiometry (OsteoSys Co., Ltd., Korea) and a region of interest was set for the hindlimbs. On the last day of the experimental period, blood samples were collected immediately after the rats were euthanized using CO_2_ gas. The skin of the hindlimb was resected to expose the muscle, and muscle thickness was measured using a digital caliper (Mitutoyo, Tokyo, Japan). The quadriceps (Quad), gastrocnemius (Gas), soleus (Sol), tibialis anterior (TA), and extensor digitorum longus (EDL) muscles were then removed, weighed, and stored at –80 °C. Relative muscle weight was calculated as absolute muscle weight (g)/body weight (g) × 100 (%). All the animal procedures were approved by the Institutional Animal Care and Use Committee (approval no. GU1-2021-IA0034) from Gachon University.

### Measurement of serum biochemistry markers

Serum biochemistry was performed using a chemistry analyzer (AU480, Beckman Coulter, Brea, CA, USA) and serum levels of interleukin (IL)-1β and tumor necrosis factor alpha (TNFα) were measured using commercial enzyme-linked immunosorbent assay (ELISA) kits (R&D systems, Minneapolis, MN, USA) according to the manufacturer’s instructions.

### Grip strength measurement

Grip strength was assessed using a grip strength meter (Ugo Basile, Comerio, Italy) in the last week of the experimental period. The rat was allowed to grasp the grid of the grip strength meter, and its tail was slowly pulled horizontally with a constant force by researchers who were blinded to the experimental groups until the rat released the grid. The test for each rat was repeated thrice at 1 min intervals. The average of the measurements was recorded and normalized to body weight.

### Hematoxylin and eosin (H&E) staining

For histological observation, Quad and Gas muscle samples were fixed with 10% neutral buffered formalin (Sigma-Aldrich, St. Louis, MO, USA) and paraffin embedded. Further, 3–4 μm sections were used for H&E staining. Pathological changes were observed using an Olympus Provis AX70 Microscope (Olympus, Tokyo, Japan) and images were captured using a DS-Ri2 camera (Nikon, Tokyo, Japan). The cross-sectional area (CSA) of at least 80 muscle fibers was quantified from three slides using ImageJ software (National Institutes of Health, USA).

### Western blot analysis

Quad muscle and C2C12 cells were homogenized and lysed in RIPA buffer (iNtRON Biotechnology, Gyeonggi-do, Korea) containing a protease and phosphatase inhibitor cocktail (Thermo Fisher Scientific, Waltham, MA, USA). The following procedures were carried out as previously described^17^. Details of the antibodies used in this study are listed in Table S4.

### Total RNA extraction and real-time RT-PCR

RNA was extracted from the muscle tissues and C2C12 cells using a total RNA extraction kit (iNtRON Biotechnology, Gyeonggi-do, Korea) according to the manufacturer’s instructions. Complementary DNA synthesis was performed using GoScript™ Reverse Transcriptase (Promega, Madison, WI, USA) according to the manufacturer’s instructions. Real-time RT-PCR was performed using TB Green Premix Ex Taq II (Takara Bio Inc., Kusatsu, Shiga) on a QuantStudio 3 Real-Time PCR System (Applied Biosystems, Foster City, CA, USA). The relative mRNA expression levels were determined using glyceraldehyde-3-phosphate dehydrogenase (GAPDH) as an internal control. The primer sequences are listed in Table S5.

### Statistical analysis

GraphPad Prism version 10 (GraphPad Software Inc., La Jolla, CA, USA) was used for statistical analysis. The results are expressed as mean ± standard deviation (SD). Parametric data were analyzed using one-way ANOVA followed by Tukey’s post hoc test or Welch’s ANOVA followed by Dunnett T3 test. Nonparametric data were analyzed using the Kruskal-Wallis test with a two-stage step-up method of Benjamini, Krieger, and Yekutieli test. At least three independent experiments were performed. A *p*-value below 0.05 was considered statistically significant.

## Supplementary information

Supplementary information

## Data Availability

All data generated or analyzed during this study are included in this published article and its supplementary information files.
