# Supplementary material for: Mealworm hydrolysate ameliorates dexamethasone-induced muscle atrophy via sirtuin 1-mediated signaling and Akt pathway
Source: NPJ Sci Food. 2025 May 13;9:72. doi: 10.1038/s41538-025-00432-9 (PMC12075696; doi:10.1038/s41538-025-00432-9)
Supplement: Supplementary file 1 — Supplementary information [file 41538_2025_432_MOESM1_ESM.pdf]

Supplementary Figures

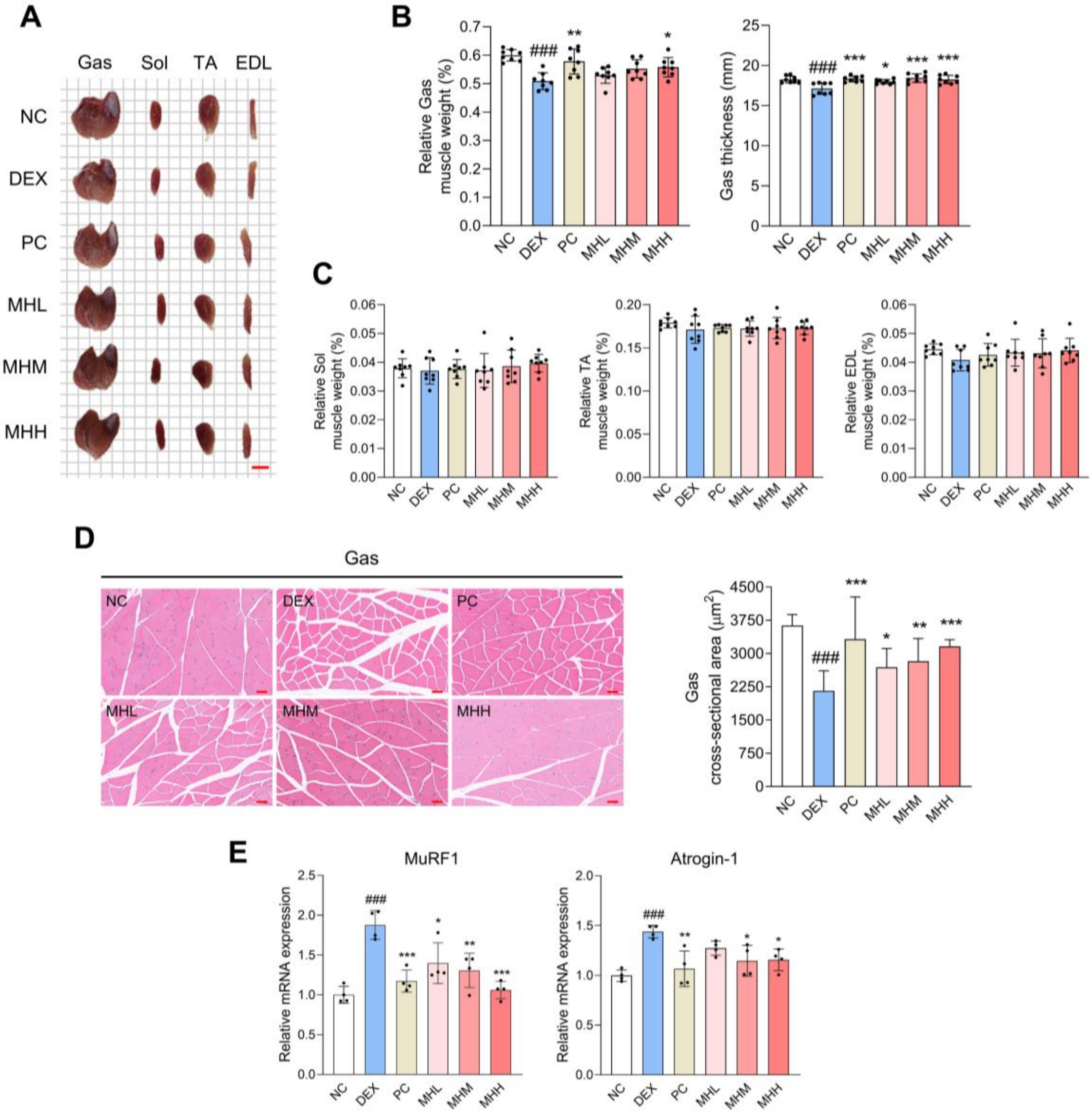

Fig. S1. Effects of MH on the Gas, Sol, TA, and EDL muscles in DEX-treated rats. Male SD rats were injected with DEX for one week, followed by oral treatment with oxymetholone (50 mg/kg/day) and MH (100, 200, and 400 mg/kg/day) for four weeks. **(A)** Representative images of the Gas, Sol, TA, and EDL muscles. The scale bar indicates 1 cm. **(B)** The relative weight and thickness of the Gas muscles (n = 8). **(C)** The relative weight of the Sol, TA, and EDL muscles (n = 8). **(D)** CSA of the Gas muscle fibers. Magnification ×200. The scale bar indicates 50 μm. **(E)** mRNA expression levels of MuRF1 and atrogin-1 in the Gas muscles. All

experiments were repeated at least three times. Data are presented as the mean ± SD. MH, mealworm hydrolysate; DEX, dexamethasone; Gas, gastrocnemius; Sol, soleus; TA, tibialis anterior; EDL, extensor digitorum longus; n.s., not significant

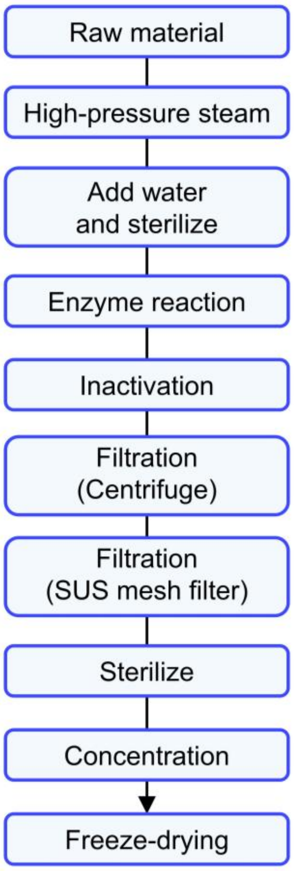

Fig. S2. Standard production procedure for MH production.

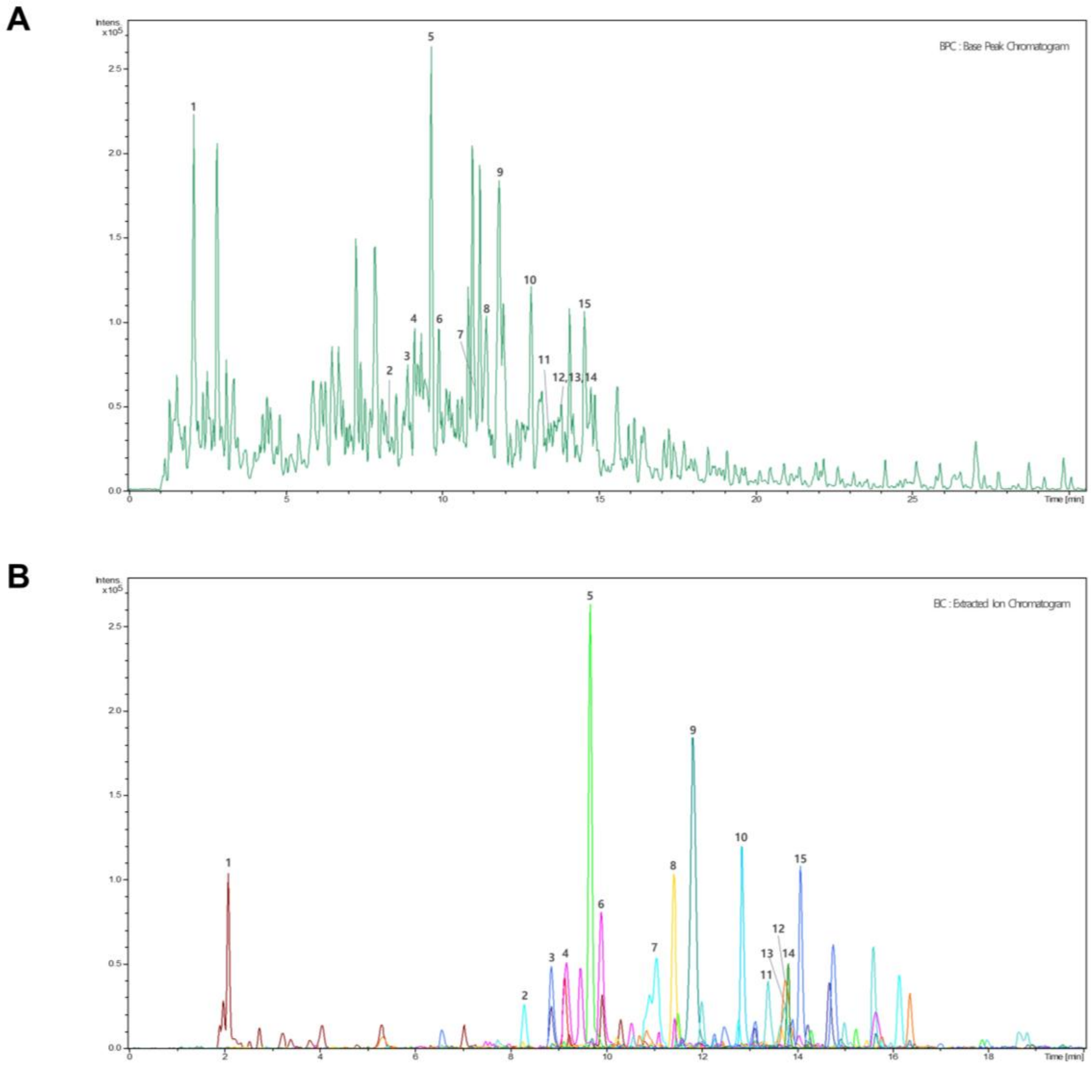

Fig. S3. Fifteen peptides were identified from MH, and peptides 5, 6, 8, 9, and 15 were selected for molecular docking analysis based on the intensity, resolution, and symmetry of the peaks. (A) LC/MS base peak chromatogram (BPC) and **(B)** extracted ion chromatogram (EIC) of MH. Peptide 1, m/z 345.23, AVR; Peptide 2, m/z 579.32, AAPLAH; Peptide 3, m/z 598.36, AAPLAR; Peptide 4, m/z 609.33, HAPVVS; Peptide5, m/z 406,79, REPLVAK; Peptide 6, m/z 428.25, AVAPA; Peptide 7, m/z 499.29, AAPAVA/AAPAAV, Peptide 8, m/z 421.21, AAPY; Peptide 9, m/z 435.24, TEAPLNPK; Peptide 10, m/z 554.33, HALLT; Peptide 11, m/z 527.32, AAPVVA/AAPVAV; Peptide 12, m/z 513.30, AAPALA/AAPAAL; Peptide 13, m/z 527.32, APVAVA/APVAAV; Peptide 14, m/z 413.26,

VAAPVAVAK; Peptide 15, m/z 456.28, VAAPV. A, alanine; E, glutamic acid; H, histidine; K, lysine; L, leucine; N, asparagine; P, proline; R, arginine; S, serine; T, threonine; V, valine; Y, tyrosine; MH, mealworm hydrolysate

Supplementary Tables

Table S1. Docking score of MH-derived peptides with SIRT1

Sequence of MH-derived peptide	SIRT1 with substrate peptide (kcal/mol)
AAPY	–14.01
AVAPA	–12.49
VAAPV	–11.35
RQPLVAK	–9.38
RQPIVAK	–10.24
TEAPLNPK	–10.21
TEAPINPK	–9.48

As leucine and isoleucine have the same mass, the two peptides, RQPLVAK (Arg-Gln-Pro-Leu-Val-Ala-Lys) and TEAPLNPK (Thr-Glu-Ala-Pro-Leu-Asn-Pro-Lys), were also evaluated as RQPIVAK (Arg-Gln-Pro-Ile-Val-Ala-Lys) and TEAPINPK (Thr-Glu-Ala-Pro-Ile-Asn-Pro-Lys), respectively. A, alanine; P, proline; Y, tyrosine; V, valine; R, arginine; Q, glutamine; L, leucine; K, lysine; I, isoleucine; T, threonine; E, glutamic acid; N, asparagine

Table S2. Serum biochemical parameters in DEX-treated rats (n = 8)

	NC	DEX	PC	MHL	MHM	MHH
AST (U/L)	101.43 ± 19.65^{NS}	104.29 ± 13.46	105.86 ± 25.40	106.86 ± 8.13	105.00 ± 11.98	100.14 ± 24.40
ALT (U/L)	36.00 ± 9.45^{NS}	35.71 ± 5.59	36.71 ± 4.39	36.43 ± 4.12	35.00 ± 5.40	34.14 ± 4.98
Total protein (g/dL)	6.16 ± 0.5^{NS}	6.26 ± 0.15	6.27 ± 0.17	6.23 ± 0.32	6.14 ± 0.26	6.23 ± 0.20
Albumin (g/dL)	4.44 ± 0.36^{NS}	4.46 ± 0.16	4.46 ± 0.16	4.39 ± 0.22	4.33 ± 0.20	4.31 ± 0.17
BUN (mg/dL)	10.74 ± 1.21^{NS}	12.34 ± 1.60	11.23 ± 1.28	10.69 ± 1.25	10.97 ± 1.41	11.04 ± 0.88
Creatinine (mg/dL)	0.41 ±0.06^{NS}	0.42 ± 0.06	0.43 ± 0.02	0.42 ± 0.05	0.39 ± 0.02	0.42 ± 0.07

Data are presented as the mean ± SD. NC, normal control; DEX, DEX-treated control; PC, positive control, DEX treatment with 50 mg/kg oxymetholone; MHL, DEX treatment with 100 mg/kg MH; MHM, DEX treatment with 200 mg/kg MH; MHH, DEX treatment with 400 mg/kg MH. AST, aspartate transaminase; ALT, alanine transaminase; BUN, blood urea nitrogen; NS, not significant

Table S3. Amino acid sequences of fifteen peptides derived from MH

Peak No.	RT (min)	Charge	m/z	Sequence
1	2.1	1	345.23	AVR
2	8.3	1	579.32	AAPLAH
3	8.8	1	598.36	AAPLAR
4	9.1	1	609.33	HAPVVS
**5**	**9.7**	**2**	**406.79**	**REPLVAK**
**6**	**9.9**	**1**	**428.25**	**AVAPA**
7	11.0	1	499.29	AAPAVA/ AAPAAV
**8**	**11.4**	**1**	**421.21**	**AAPY**
**9**	**11.8**	**2**	**435.24**	**TEAPLNPK**
10	12.8	1	554.33	HALLT
11	13.4	1	527.32	AAPVVA/ AAPVAV
12	13.7	1	513.30	AAPALA/ AAPAAL
13	13.7	1	527.32	APVAVA/ APVAAV
14	13.8	2	413.26	VAAPVAVAK
**15**	**14.0**	**1**	**456.28**	**VAAPV**

Peptides 5, 6, 8, 9, and 15 were employed in the molecular docking study. A, alanine; E, glutamic acid; H, histidine; K, lysine; L, leucine; N, asparagine; P, proline; R, arginine; S, serine; T, threonine; V, valine; Y, tyrosine

Table S4. List of antibodies used for western blotting

Antibody	Supplier	Source	Cat. No.
MyHC	R&D systems	Mouse	MAB4470
SIRT1	Cell Signaling Technology	Rabbit	9475
p-Akt (T308)	Cell Signaling Technology	Rabbit	13038
Akt	Cell Signaling Technology	Rabbit	9272
p-FOXO3a (S253)	Cell Signaling Technology	Rabbit	9466
FOXO3a	Cell Signaling Technology	Rabbit	2497
MuRF1	Abcam	Rabbit	ab183094
Atrogin-1	Abcam	Rabbit	ab168372
p-mTOR (S2448)	Cell Signaling Technology	Rabbit	5536
mTOR	Cell Signaling Technology	Rabbit	2972
p-p70S6K (T389)	Cell Signaling Technology	Rabbit	9205
p70S6K	Cell Signaling Technology	Rabbit	9202
p-4E-BP1 (T37/46)	Cell Signaling Technology	Rabbit	2855
4E-BP1	Cell Signaling Technology	Rabbit	9644
Cleaved caspase-3	Cell Signaling Technology	Rabbit	9661
Bax	Cell Signaling Technology	Rabbit	2772
Bcl-2	Abcam	Rabbit	ab182858
p-AMPK (T172)	Cell Signaling Technology	Rabbit	2535
AMPK	Cell Signaling Technology	Rabbit	2532
PGC-1α	Bioss	Rabbit	bs-7535R
UCP3	Abcam	Rabbit	ab180643
GAPDH	Cell Signaling Technology	Rabbit	5174
Anti-Rabbit IgG, HRP Conjugate	Promega	Goat	W4011
Anti-Mouse IgG, HRP Conjugate	Promega	Goat	W4021

Table S5. List of primer sequences used for real-time RT-PCR

Genes	Accession Number	Primer Sequence (5′–3′)	Product length
MuRF1	NM_080903.2	F: GCCATCCTGGACGAGAAGAAG<br>R: AGCGGCTTGGCACTCAAG	194
Atrogin-1	NM_133521.2	F: CCAGAGAGTCGGCAAGTC<br>R: CAGGTCGGTGATCGTGAG<br>R: GCGTTGCCAGAAGTGAAGCCA	142
GAPDH	NM_017008.4	F: CTGCACCACCAACTGCTTAG<br>R: GGATGCAGGGATGATGTTCT	178

Original blots

Fig. 1E

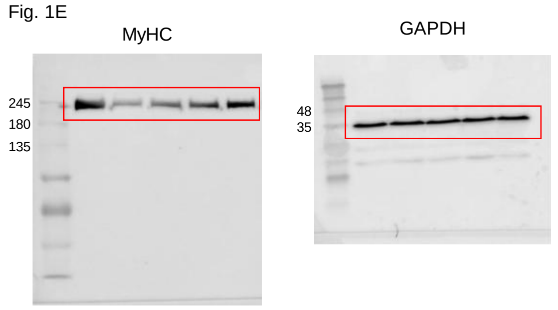

Fig. 2B

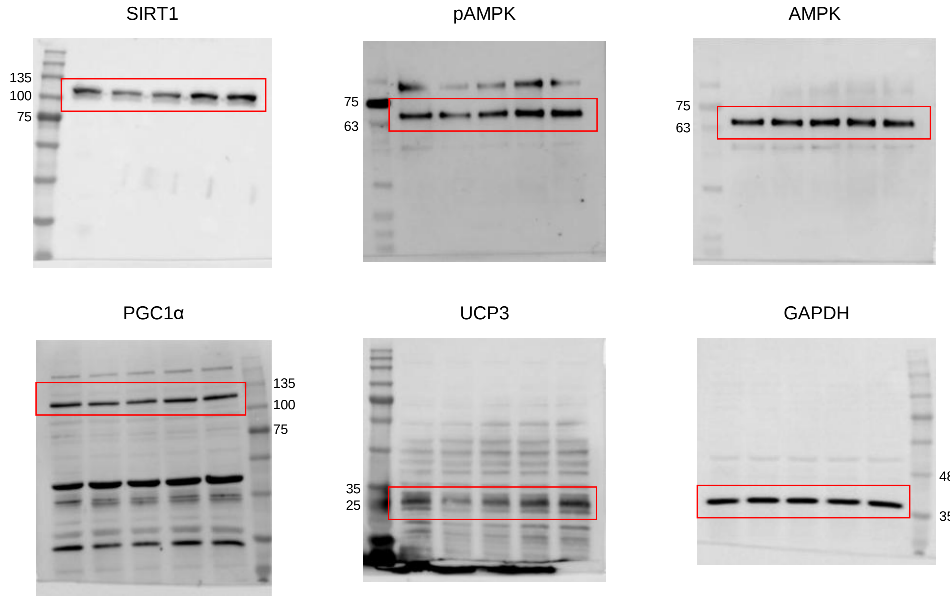

Fig. 2C

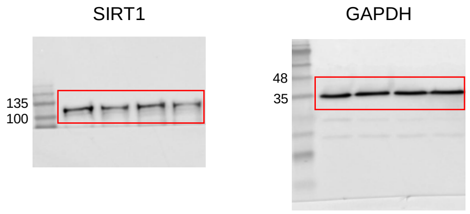

Fig. 3A

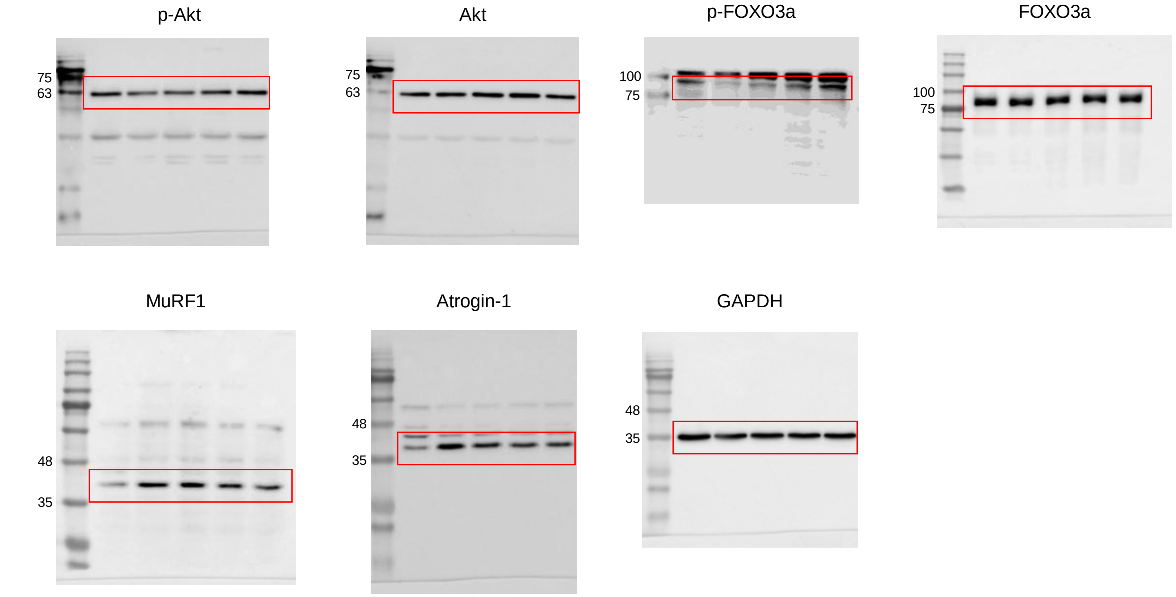
p-Akt
Akt
p-FOXO3a
FOXO3a
75
63
75
63
100
75
100
75
MuRF1
Atrogin-1
GAPDH
48
35
48
35
48
35

Fig. 3B

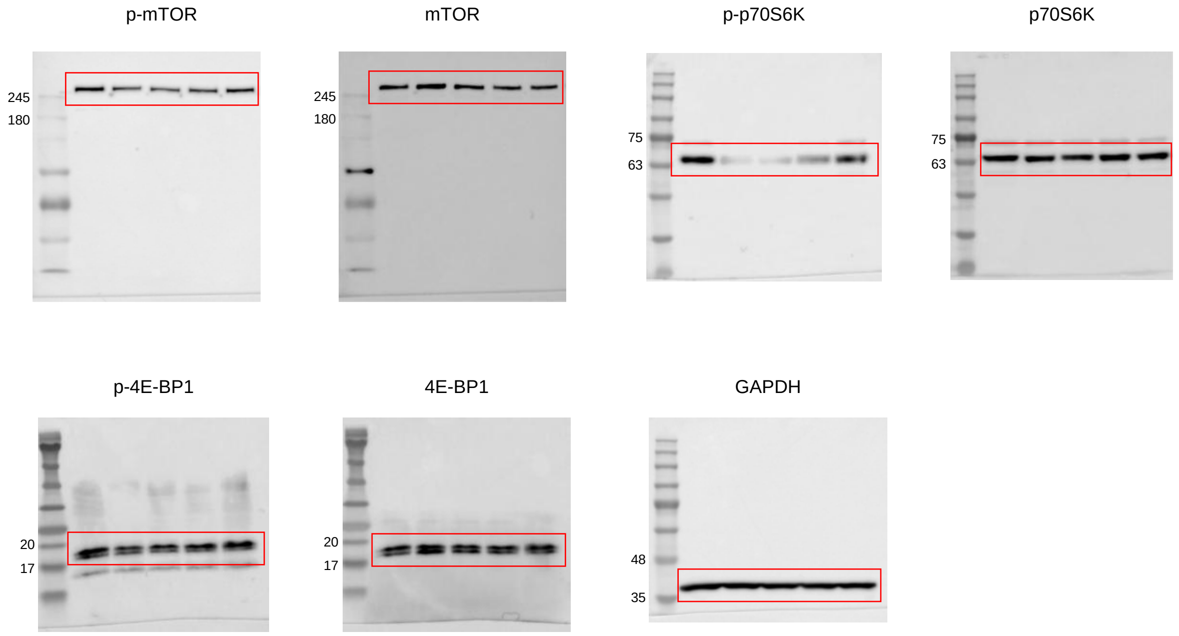
p-mTOR
mTOR
p-p70S6K
p70S6K
245
180
245
180
75
63
75
63
p-4E-BP1
4E-BP1
GAPDH
20
17
20
17
48
35

Fig. 3C

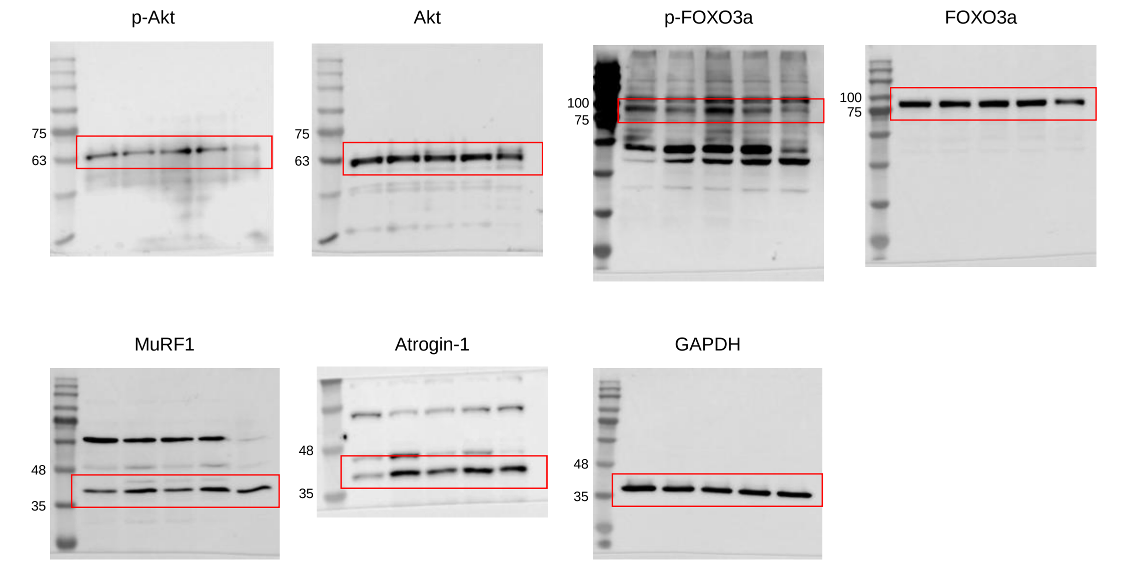
p-Akt
75
63
Akt
75
63
p-FOXO3a
100
75
FOXO3a
100
75
MuRF1
48
35
Atrogin-1
48
35
GAPDH
48
35

Fig. 3D

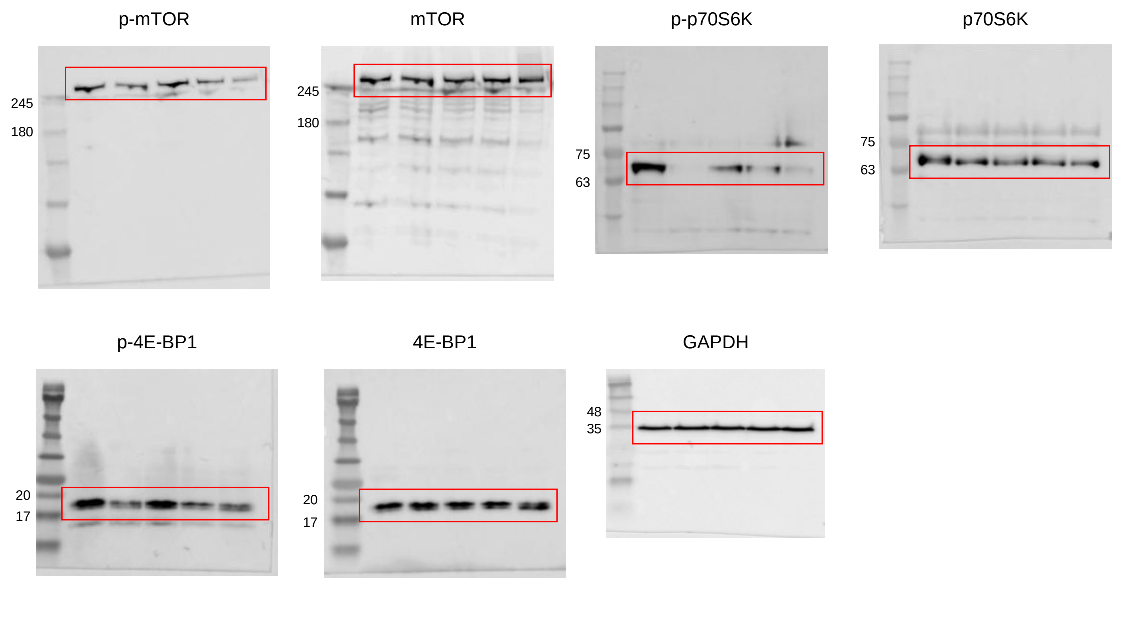
p-mTOR
245
180
mTOR
245
180
p-p70S6K
75
63
p70S6K
75
63
p-4E-BP1
20
17
4E-BP1
20
17
GAPDH
48
35

Fig. 4A

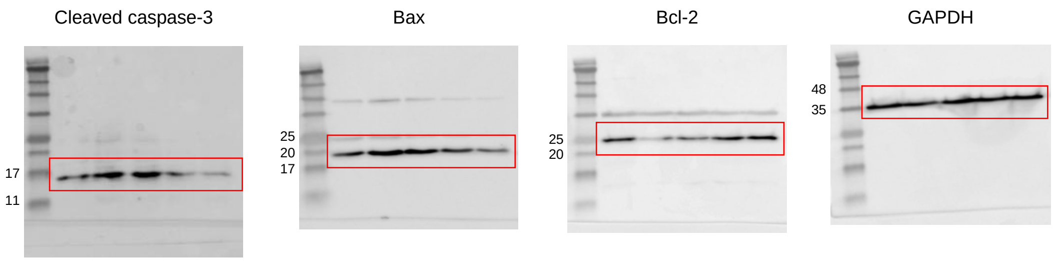
Cleaved caspase-3
17
11
Bax
25
20
17
Bcl-2
25
20
GAPDH
48
35

Fig. 6B

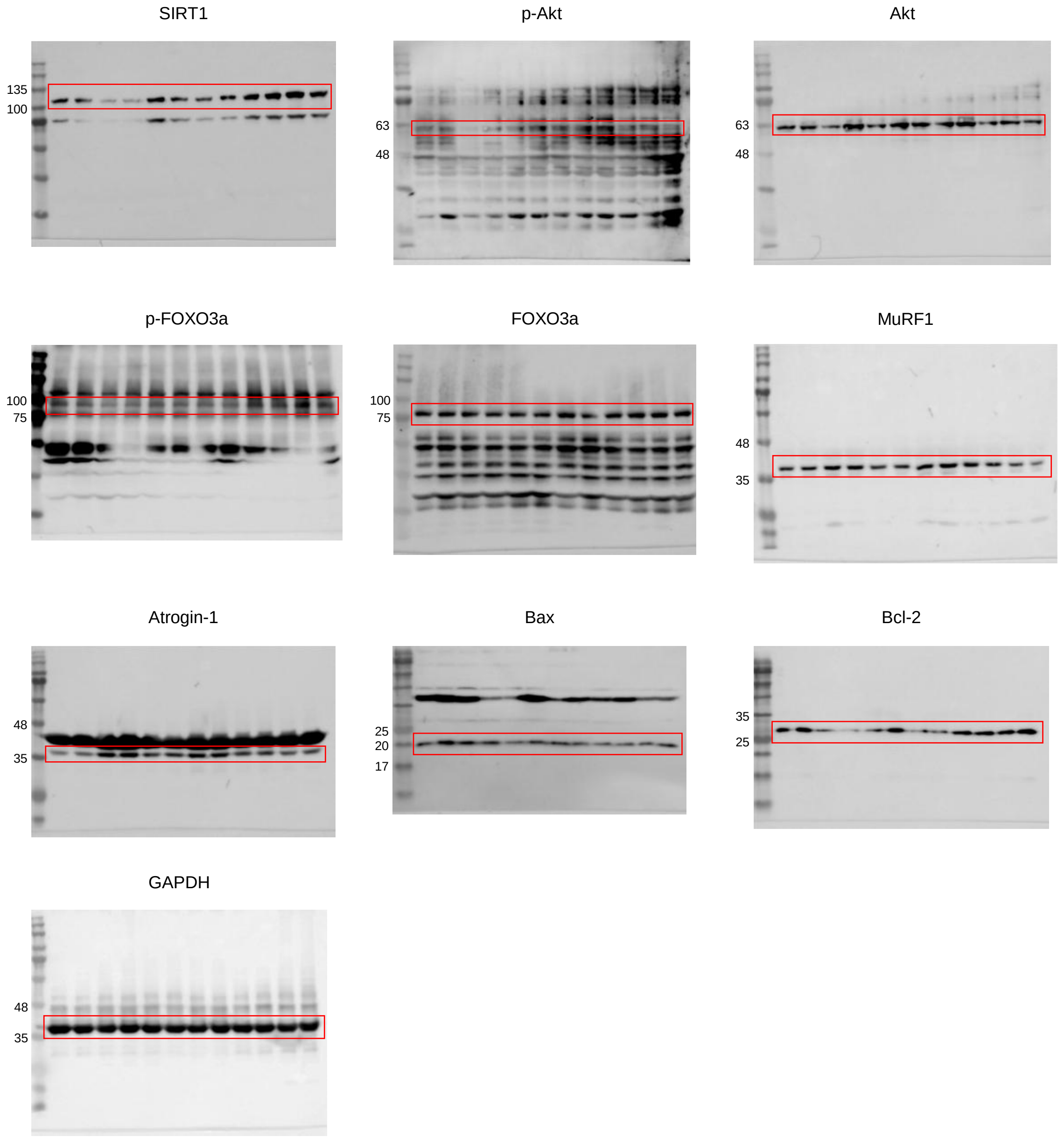
SIRT1
135
100
p-Akt
63
48
Akt
63
48
p-FOXO3a
100
75
FOXO3a
100
75
MuRF1
48
35
Atrogin-1
48
35
Bax
25
20
17
Bcl-2
35
25
GAPDH
48
35

The ARRIVE Essential 10: Compliance Questionnaire

Use this questionnaire to evaluate how well a manuscript complies with the ARRIVE Essential 10. It can be applied to any manuscript describing comparative experiments in living animals, by assessors such as journal staff, editors, or peer reviewers.

Item		Question(s)	Answers
1	Study Design	Are all experimental and control groups clearly identified?	☒ Yes, for at least one experiment<br>☐ No
		Is the experimental unit (e.g. an animal, litter or cage of animals) clearly identified?	☒ Yes, for at least one experiment<br>☐ No
2	Sample Size	Is the exact number of experimental units in each group at the start of the study provided (e.g. in the format '*n*=')?	☒ Yes, for at least one experiment<br>☐ No
		Is the method by which the sample size was chosen explained?	☒ Yes, for at least one experiment<br>☐ No
3	Inclusion & Exclusion Criteria	Are the criteria used for including and excluding animals, experimental units, or data points provided?	☒ Yes, for at least one experiment<br>☐ No
		Are any exclusions of animals, experimental units, or data points reported, or is there a statement indicating that there were no exclusions?	☒ Yes, for at least one analysis<br>☐ No
4	Randomisation	Is the method by which experimental units were allocated to control and treatment groups described?	☒ Yes, for at least one experiment<br>☐ No
5	Blinding	Is it clear whether researchers were aware of, or blinded to, the group allocation at any stage of the experiment or data analysis?	☒ Yes, for at least one experiment<br>☐ No
6	Outcome Measures	For all experimental outcomes presented, are details provided of exactly what parameter was measured?	☒ Yes, for at least one experiment<br>☐ No
7	Statistical Methods	Is the statistical approach used to analyse each outcome detailed?	☒ Yes, for at least one analysis<br>☐ No
		Is there a description of any methods used to assess whether data met statistical assumptions?	☒ Yes, for at least one analysis<br>☐ No<br>☐ Not applicable
8	Experimental Animals	Are all species of animal used specified?	☒ Yes, for at least one experiment<br>☐ No
		Is the sex of the animals specified?	☒ Yes, for at least one experiment<br>☐ No<br>☐ Not applicable to species
		Is at least one of age, weight or developmental stage of the animals specified?	☒ Yes, for at least one experiment<br>☐ No
9	Experimental Procedures	Are both the timing and frequency with which procedures took place specified?	☒ Yes, for at least one experiment<br>☐ No
		Are details of acclimatisation periods to experimental locations provided?	☒ Yes, for at least one experiment<br>☐ No
10	Results	Are descriptive statistics for each experimental group provided, with a measure of variability (e.g. mean and SD, or median and range)?	☒ Yes, for at least one experiment<br>☐ No<br>☐ Not applicable to the type of data collected
		Is the effect size and confidence interval provided?	☐ Yes, for at least one experiment<br>☐ No<br>☒ Not applicable to the type of analysis used

ARRIVE

NC3Rs National Centre for the Replacement Refinement & Reduction of Animals in Research

Notes on questionnaire design

The ARRIVE guidelines are a useful resource for authors preparing manuscripts describing animal research, and also provide a framework to evaluate the transparency of those manuscripts. To assess reporting quality, numerous studies have in the past sought to operationalise reporting guidelines (including ARRIVE). Typically, this involves scoring a manuscript's degree of compliance with guideline items in a binary fashion (e.g. an item is either not reported or reported) [1-3], a graded fashion (e.g. not, partially, or completely reported) [4,5], or a combination of the two [6].

This questionnaire has been designed to be as concise and user-friendly as possible. The number of questions used to assess a manuscript's compliance has been kept to a minimum, and in most cases each question is designed to be answered in a binary fashion. Compliance with some Essential 10 sub-items is inherently impossible to judge in this way, instead requiring a subjective judgement on the level of detail provided. For this reason, not all sub-items are represented by a question in this questionnaire.

To facilitate binary answers, it has been necessary to identify the minimum information in a manuscript sufficient to comply with each question. The strengths of this approach include the relatively short length of the questionnaire (and the correspondingly low time burden of using it), and the avoidance of ambiguity that would arise from a graded answering system, in which an intermediate score (e.g. 'partially/insufficiently reported') could denote a number of distinct deficiencies in compliance with an item (e.g. either only part of the item was complied with, or only the reporting of some experiments in the manuscript complied with the item.)

Limitations of this approach centre on the necessity to identify the minimum information sufficient to comply with each question. In some cases, this has resulted in questions that require a guideline sub-item's criteria to have been fulfilled in the reporting of only one experiment in a manuscript. As a result, not all experiments in a manuscript may be described in a way that fulfils that criterion, despite the manuscript being considered to comply with the guidelines overall.

References

1. Hair *et al* (2020). *Res Integ Peer Rev*. doi: 10.1186/s41073-019-0069-3
2. Tihanyi *et al* (2019). *J Surg Res*. doi: 10.1016/j.jss.2018.10.038
3. Zhao *et al* (2020). *BMC Vet Res*. doi: 10.1186/s12917-020-02664-1
4. Han *et al* (2017). *Plos One*. doi: 10.1371/journal.pone.0183591
5. Chatzimanouil *et al* (2019). *J Am Soc Nephrol*. doi: 10.1681/ASN.2018050515
6. Leung *et al* (2018). *Plos One*. doi: 10.1371/journal.pone.0197882
